# Pregnancy-related Hemophagocytic Lymphohistiocytosis Associated with Herpes Simplex Virus-2 Infection: A Diagnostic Dilemma

**DOI:** 10.7759/cureus.2352

**Published:** 2018-03-20

**Authors:** M. Farhan Nasser, Shorabh Sharma, Elizabeth Albers, Sapna Sharma, Anurag Duggal

**Affiliations:** 1 Internal Medicine, Cleveland Clinic Akron General; 2 Internal Medicine, SBH Health System; 3 Medical Student, Northeast Ohio Medical University (NEOMED); 4 Internal Medicine, Mahatma Gandhi Mission Institute of Health Sciences; 5 Infectious Diseases, Cleveland Clinic Akron General

**Keywords:** hemophagocytic lymphohistiocytosis, hsv, pregnancy

## Abstract

Hemophagocytic lymphohistiocytosis (HLH) is a severe inflammatory disorder characterized by the uncontrolled proliferation of lymphocytes and histiocytes with hemophagocytic activity in the bone marrow. To our knowledge, there have been a few reported cases of pregnancy-related HLH. This case highlights the importance of considering HLH in a pregnant woman along with other diagnoses, such as HELLP (which stands for hemolysis, elevated liver enzyme levels, and low platelet levels) syndrome and hemolytic anemias. It points to the challenges of diagnosing and managing pregnancy-related HLH due to a similarity in presentation with other conditions.

## Introduction

Hemophagocytic lymphohistiocytic syndrome (HLH) is a rare life-threatening disorder that results from the uncontrolled activation of the immune system.

HLH can be classified as primary or secondary. Primary HLH, which is also known as familial HLH, is autosomal recessive and usually presents in childhood. Secondary HLH may result due to immunological activation by malignancies, autoimmune disorders or infections, of which viruses, particularly Epstein Barr Virus (EBV), is the most common [1].

To our knowledge, there have been very few reported cases of HLH in pregnancy. Here, we present a case of HLH associated with herpes simplex virus-2 (HSV) in a patient during her third trimester of pregnancy.

## Case presentation

A 36-year-old woman, gravida 7 para 2 abortus 4, presented to the hospital at 31 weeks' gestation with symptoms suggestive of an upper respiratory infection. An examination revealed splenomegaly. Initial laboratory work-up revealed a platelet count of 118 x 109/L and transaminitis with a serum aspartate transaminase (AST) of 130 U/L and serum alanine transaminase (ALT) of 87 U/L. Serial lab work showed progressively worsening transaminitis and thrombocytopenia, as a result of which the patient was rushed to an emergency C-section due to concern for pre-eclampsia. The patient delivered a five pound live born female infant. The day following surgery, she developed a blanching erythematous rash on her face and chest. She started developing fevers of up to 40 C on successive days. Blood cultures, urine cultures, viral antibody titers for herpes simplex virus 1/2 (HSV), cytomegalovirus (CMV), Epstein Barr Virus (EBV), parvovirus, and the viral hepatitis panels were all negative. Her liver enzymes continued to increase, along with a decreasing hemoglobin of 9 g/dl and a platelet level of 73,000/L. Ferritin and lactate dehydrogenase (LDH) were checked, which were found to be elevated at 5296.8 ng/ml and 2284 U/L. In light of suspicion for HLH, she was started on dexamethasone. She then underwent a bone marrow biopsy, which revealed “normocellular marrow with trilineage hematopoiesis and evidence of hemophagocytosis.” Subsequently, the baby tested positive for HSV and the patient was then started on acyclovir. Repeat HSV 2 IgM/IgG were positive. Over the course of her days up to discharge, her rash, transaminitis, and thrombocytopenia had all resolved. The patient was then discharged with a two-week prescription for acyclovir. It was found that the baby had unfortunately died of HSV encephalitis 4 days after her birth.

## Discussion

The diagnosis of HLH requires the detection of an HLH-associated mutation or the presence of at least five of the following eight criteria: fever > 38.5C, ferritin > 500 ng/ml (levels >3000 ng/ml are more suggestive), two peripheral blood cytopenias, hypertriglyceridemia and/or hypofibrinogenemia, hemophagocytosis in the bone marrow, spleen, lymph node or liver, splenomegaly, low or absent natural killer (NK) cell activity, or elevated soluble CD 25. In the case of an inconclusive bone marrow biopsy, other organs can be evaluated. Patients who have an HLH-associated mutation do not need to fulfill the diagnostic criteria [2]. As early initiation of treatment can potentially be lifesaving, the diagnostic criteria do not need to be fulfilled prior to starting therapy.

Reactive HLH is associated with a variety of microbial agents, more commonly, viruses such as EBV, cytomegalovirus, human herpesvirus-8 (HHV-8), HIV, influenza, and parvovirus. EBV is the most common causative agent [1]. There are a few cases of HLH that are reported to be secondary to HSV-2.

The diagnosis and management of HLH during pregnancy are complicated, as the presentation mimics that of HELLP (hemolysis, elevated liver enzymes, low Platelets) syndrome and the acute fatty liver of pregnancy. The persistence of anemia, thrombocytopenia, and elevated liver enzymes beyond the delivery of the baby made HELLP syndrome unlikely [3]. It is imperative to differentiate HLH from similarly presenting conditions, such as HELLP, due to their different complications and therapeutic implications.

The goal of treatment is to suppress the inflammatory response and treat the underlying cause [3-4]. Many treatment options, such as rituximab and etoposide, which have been listed in previous literature are either not safe in pregnancy or their safety has not been established [5]. As a result, the options for the treatment of HLH in pregnancy are limited. Treatment options, such as intravenous immunoglobulin and cyclosporine, which have been successful, were not tried on our patient. Our case is one that was successfully treated with acyclovir in combination with high-dose steroids. As high dose steroids can be safely administered in pregnancy, they will always remain one of the first-line treatments to be used in HLH in pregnancy [3].

## Conclusions

In conclusion, HLH should be part of the differential diagnoses in a patient with splenomegaly, transaminitis, anemia, and thrombocytopenia along with persistent fevers. HLH can be associated with many diseases and its prognosis is usually determined by the underlying disease, as a result of which, it is equally important to diagnose the secondary cause. An early recognition of the symptoms of HLH and the initiation of treatment will improve outcomes; however, the treatment options for pregnancy-induced HLH need further evaluation.
